# Evaluation of the Suture Pull-Out Technique in Anterior Cruciate Ligament Avulsion Fractures at Different Weeks Post-fracture

**DOI:** 10.7759/cureus.69126

**Published:** 2024-09-10

**Authors:** Tapan Kumar Das, Manjit Dash, Aniruddh Dash, Jitendra Mishra, Nego Zion

**Affiliations:** 1 Orthopaedics, Siksha 'O' Anusandhan, Bhubaneswar, IND

**Keywords:** acl avulsion, anterior cruciate ligament (acl), arthroscopic suture pull-out technique, arthroscopy knee, tibial spine avulsion

## Abstract

Background

Anterior cruciate ligament (ACL) avulsion fractures often necessitate surgical intervention, with various fixation techniques available. Among these, the arthroscopic suture pull-out technique has gained attention as a promising approach. However, the influence of surgical timing on patient outcomes remains insufficiently studied. This study aims to evaluate the efficacy of the arthroscopic suture pull-out technique for ACL tibial avulsion fractures and assess how the timing of surgical intervention affects functional outcomes.

Methodology

This study was conducted at our hospital from November 2020 to October 2022. A total of 17 patients aged 21 to 41 years with isolated ACL avulsion fractures and no additional injuries or osteoarthritis were included. Participants were divided into three groups based on the interval from injury to surgery (one, two, or three weeks). The surgical procedure involved the suture pull-out technique. Postoperative management included immobilization, isometric exercises, and gradual weight-bearing. Functional outcomes were assessed using the Lysholm score, International Knee Documentation Committee (IKDC) score, Tegner Activity Scale, and Lachman test at 6, 10, and 14 months, along with radiological evaluations.

Results

Significant functional improvements were observed in all groups, with postoperative Lysholm, IKDC, and Tegner scores showing notable enhancements compared to preoperative values. No significant differences were found among the groups regarding the timing of surgery, indicating that delays within the first three weeks did not adversely impact outcomes. Most patients achieved a full range of motion. Two minor postoperative complications were reported, namely, one case of arthrofibrosis and one case of persistent laxity.

Conclusions

The arthroscopic suture pull-out technique is effective and reliable for ACL tibial avulsion fixation. The timing of surgery within the first three weeks does not significantly affect functional outcomes. Future research with larger sample sizes and longer follow-ups is recommended to further validate these findings and optimize surgical strategies.

## Introduction

Anterior cruciate ligament (ACL) avulsion fractures can occur due to various mechanisms. In children, these injuries typically result from knee bending combined with inward tibial rotation and are often isolated, without other knee injuries. In adults, ACL avulsion fractures are usually caused by severe hyperextension, such as in motor vehicle collisions, and are frequently associated with additional injuries, including damage to the medial collateral ligament (MCL) and posterior cruciate ligament (PCL) [1,2]. The incidence of isolated ACL avulsion fractures is approximately 3 per 100,000 annually [3]. The Meyers and McKeever classification system outlines four distinct subtypes of tibial spine fractures, which guide treatment selection [4]. Type I involves a minimally displaced fragment. In Type II, the fracture fragment is elevated anteriorly, while Types III and IV show complete separation of the fragment from the tibia. Type IV also features comminution of the fragment [2]. Some studies have further subdivided Type III injuries into the following two subtypes: Type IIIa, where the fragment is simply separated, and Type IIIb, where the fragment is not only separated but also rotated, or the fracture extends beyond the ACL insertion area [5]. Type I injuries are typically treated conservatively [4]. However, the treatment for Type II fractures remains controversial [4,6]. For Types III and IV fractures, various surgical techniques are employed, including retrograde wires, cancellous screws, suture anchors, suture bridges, and Kirschner wires with tension band wiring [7]. The suture pull-out technique has shown promising results for ACL avulsion fractures, with studies suggesting its effectiveness over other methods, such as screw fixation [8]. However, the timing of surgical intervention remains a critical factor influencing patient outcomes, and this aspect has not been extensively explored in the literature.

This study aims to evaluate the arthroscopic suture pull-out technique in ACL tibial avulsion fractures, with a specific focus on the timing of surgical intervention. We intend to assess the restoration of knee range of motion; compare subjective functional outcomes measured by the International Knee Documentation Committee (IKDC) score, Lysholm score, Tegner Activity Scale, and Lachman test; and identify complications following the suture pull-out technique. By addressing the timing of surgery, this study seeks to provide insights into the optimal timing for surgical repair to achieve the best functional recovery.

## Materials and methods

This prospective study was conducted at our hospital from November 2020 to October 2022. A total of 17 patients aged 21-41 years with ACL avulsion fractures, who were evaluated clinically and radiologically, were included. The demographic details and mode of injury were documented. The study population was categorized into the following three groups based on the time elapsed between the injury and surgical intervention: Group 1 (within one week post-injury/1-7 days), Group 2 (within two weeks post-injury/8-14 days), and Group 3 (within three weeks/15-21 days). Patients with associated injuries such as medial collateral ligament, posterior cruciate ligament, lateral collateral ligament, or posterolateral corner injuries, as well as those with osteoarthritis, were excluded. Physical examinations included evaluating swelling, deformity, tenderness, and range of motion, supplemented by specific physical tests. Radiographs confirmed the diagnosis and determined the location, type, and extent of the fracture. Preoperative MRI was performed in all cases of ACL avulsions to rule out other ligament injuries and meniscal damage, as well as to document the integrity of the ACL fibers. Radiographic examinations were conducted at the time of injury, post-surgery, and at the last follow-up. Subjective functional outcomes were assessed, and Lysholm, IKDC, and Tegner scores were calculated at minimum follow-ups of 6, 10, and 14 months. Any complications were documented.

Surgical technique

Under spinal anesthesia, patients were positioned supine with a tourniquet applied to the upper thigh. The knee and calf were positioned at a 90° angle using a leg holder. Prophylactic antibiotics were administered, and after skin preparation and draping, anterolateral and anteromedial portals were marked. A tourniquet (250-320 mmHg) was inflated. A standard arthroscopic system was used with an anteromedial working portal, an anterolateral visual portal, and an accessory central portal. The hematoma around the fracture site was cleared, and obstructing tissues were removed to aid in fragment reduction. Diagnostic arthroscopy was performed to check for additional knee joint injuries. The fragment’s crater was debrided with an arthroscopic shaver. Two separate Fibertape sutures were chosen, and using a Lasso with a wire loop, sutures were passed through the ACL (Video 1). A longitudinal incision was made on the anteromedial tibia 4 cm below the joint line. An ACL tibial tunnel of 4 mm in size was drilled. A Beath pin was introduced through the tibial tunnel, and the four ends of the two Fibertapes were retrieved at the tibial end of the tunnel. While the arthroscope was inside the joint, fracture reduction was checked, and anatomic reduction was achieved with a probe under fluoroscopic guidance. Both Fibertapes were tied together over a suture disc with the knee flexed at 20°. Stability was assessed through the knee’s range of motion, followed by joint irrigation and closure of the surgical wound.

**Video 1:** Arthroscopic anterior cruciate ligament avulsion fixation.

Postoperatively, the knee was immobilized in a fully extended position for two weeks. Early postoperative care included isometric quadriceps exercises. Postoperative X-rays verified fracture reduction, checked for alignment, and monitored the healing process. This step was crucial for identifying complications and allowing for timely intervention. Patients ambulated with protected weight-bearing using axillary crutches or a universal walker until four weeks postoperatively, after which partial weight-bearing was allowed. Full weight-bearing without crutches was permitted following radiological evidence of complete fracture union.

Statistical analysis

The collected data were analyzed statistically using one-sample tests and paired-sample tests. These analyses were conducted to assess the significance of changes in clinical outcomes, including knee range of motion, pain status, and functional scores over time. Statistical significance was set at p-values <0.05.

## Results

A total of 17 patients were evaluated and monitored postoperatively for an average duration of 12.43 months, with a follow-up range of 12 to 15 months. The mean age of the study participants was 28.47 years, with a range of 21 to 41 years. Of the total study participants, 11 were male and 6 were female. Male patients predominated over female patients in this study. Among the 17 patients, sports-related injuries were the most frequent, accounting for nine (52.9%) patients. This was followed by injuries from road traffic accidents (RTAs), which were seen in seven (41.2%) patients. Self-falls were the least common, occurring in only one (5.9%) patient (Table 1).

**Table 1:** Mode of injury.

Mode of injury	Number of patients (n = 17)	Percentage
Sports injuries	9	52.9%
Road traffic accidents	7	41.2%
Self-fall	1	5.9%

Most patients in the study did not present with any associated injuries, allowing for a focused assessment of ACL avulsion fractures. According to the Meyers and McKeever classification, two (11.8%) patients were classified as Type 2, while the majority, eight (47.1%) patients, were classified as Type 3, followed by seven (41.2%) patients as Type 4. The time between injury and surgical intervention varied from one to three weeks. Of the 17 patients, four (23.5%) patients in Group 1 underwent surgery in the first week after injury, six (35.3%) patients in Group 2 were operated on in the second week, and seven (41.2%) patients in Group 3 were operated on in the third week. Preoperative Lysholm, IKDC, and Tegner Activity Scale scores were recorded, along with Lachman test findings and knee range of motion. The mean surgical duration was 130 minutes (range = 130-210 minutes), reflecting the meticulous nature of the surgical procedure to achieve optimal ligament fixation and bone healing. The characteristics of the patients are presented in Table 2.

**Table 2:** Patient characteristics. RTA: road traffic accident

Age	Sex	Injury mechanism	Mode of trauma	Time from injury to surgery in weeks	Operative time (hours)	Follow-up months
29	Male	Knee twist	Sports injury	1	2.10	12 months
35	Female	Fall	Domestic fall	3	1.35	12 months
24	Female	Knee twist	Sports injury	3	1.30	12 months
37	Male	Fall	RTA	2	1.40	12 months
41	Male	Fall	RTA	1	2	12 months
22	Male	Knee twist	Sports injury	2	1.45	12 months
39	Male	Knee twist	Sports injury	2	1.45	12 months
21	Female	Knee twist	Sports injury	3	1.20	12 months
26	Female	Fall	RTA	3	1.30	12 months
27	Male	Fall	RTA	1	2	12 months
27	Male	Fall	RTA	1	2.10	12 months
29	Male	Knee twist	Sports injury	3	1.35	12 months
24	Female	Knee twist	Sports injury	3	1.30	12 months
22	Male	Knee twist	Sports injury	3	1.20	12 months
21	Female	Knee twist	Sports injury	2	1.30	12 months
28	Male	Fall	RTA	2	1.40	12 months
32	Male	Fall	RTA	2	1.40	12 months

Postoperative functional outcome scores were recorded at the final follow-up. The Lysholm score had a preoperative average of 60.11, and the final mean score was 90.17, suggesting improved knee function after surgical intervention. However, the three groups showed no significant statistical difference (Table 3). The IKDC score had a preoperative mean of 53.20, which improved postoperatively to 78.57. There were no statistically significant differences between the three groups (Table 4). The mean Tegner Activity Scale score recorded preoperatively was 2.7, indicating a relatively low level of activity. Postoperatively, the score increased to 6.5, reflecting a significant improvement in the ability to perform physical activities following the surgical intervention. However, there were no statistically significant differences between the three groups (Table 5).

**Table 3:** Lysholm score.

		Lysholm score (mean)	Standard deviation
Group 1	Preoperative	57	4.243
	Final follow-up	91.5	3.707
Group 2	Preoperative	57.33	3.786
	Final follow-up	88.33	4.509
Group 3	Preoperative	66	5.875
	Final follow-up	90.67	1.528

**Table 4:** International Knee Documentation Committee (IKDC) score.

		IKDC (mean)	Standard deviation
Group 1	Preoperative	54.55	7.2832
	Final follow-up	77.55	5.7276
Group 2	Preoperative	53.26	3.9000
	Final follow-up	80.43	4.0624
Group 3	Preoperative	51.8	4.2572
	Final follow-up	77.73	5.2918

**Table 5:** Tegner Activity Scale score

		Tegner Activity Scale ( mean)	Standard deviation
Group 1	Preoperative	2.83	7.2832
	Final follow-up	6.45	5.7276
Group 2	Preoperative	2.46	3.9000
	Final follow-up	6.89	4.0624
Group 3	Preoperative	2.75	4.2572
	Final follow-up	6.9	5.2918

Knee joint stability, examined using the Lachman test preoperatively and at the final follow-up, showed that all patients had a lax knee preoperatively, and all patients except one had a stable knee at the final follow-up. The one patient with grade 1 laxity postoperatively was in Group 3 who initially had sustained an RTA and was managed conservatively for the postoperative laxity. At the final follow-up, 15 patients achieved a full range of motion in the knee joint. Two patients exhibited limitations: one had an extension lag of 10° compared to the other side, and the other had difficulty achieving 30° of terminal knee flexion. One patient among the 17 had knee stiffness due to arthrofibrosis and underwent arthroscopic arthrolysis. Functional outcomes demonstrated no statistically significant variation across the three groups.

## Discussion

This prospective cohort study at our hospital from November 2020 to October 2022 evaluated the efficacy of the arthroscopic suture pull-out technique for ACL tibial avulsion fixation. We aimed to assess the range of motion, functional outcomes, and complications, with a specific focus on the impact of injury-to-surgery time on these outcomes. This area has not been extensively covered in previous studies [9,10]. RTAs were the leading cause of ACL avulsion among skeletally mature patients in previous studies [4]. However, in our study, sports injuries were the predominant cause, affecting nine (53%) patients, followed by RTAs in seven (41%) patients, and self-falls in one (6%) patient. In this study, the injury mechanisms predominantly included knee twists and falls, which are common occurrences in both athletic and non-athletic populations. Male predominance was noted in our study (male:female = 65:35), similar to previous studies [11-13].

Patients were divided into three groups based on post-injury duration. Group 3 had the highest number (7 out of 17 patients) compared to the other two groups. Fracture types of the patients in these three groups were classified based on the Meyer and McKeever system, showing that 8 out of 17 patients (47.1%) had Type 3 fractures. A similar predominance of Type 3 fractures was noted in other studies by Seon et al. [11], Pan et al. [12], and Callanan et al. [14].

Managing displaced fractures is a subject of debate, with various treatment approaches proposed. Options for fixing ACL avulsions include cancellous screws, staples, sutures, K-wires, and bioabsorbable suture anchors [15]. The conservative approach has its own complications, such as extended immobilization, knee stiffness, and lingering instability. Open fixation methods can lead to varying levels of knee stiffness due to the extensive dissection involved and often necessitate a prolonged period of immobilization. Ahn et al. [16] reported that using a modified pull-out suturing technique for arthroscopic reduction in displaced tibial spine ACL avulsion fractures led to a high union rate in both acute and chronic cases. We employed the arthroscopic suture pull-out technique for treating acute ACL avulsion patients.

In our study, the average postoperative Lysholm score was highest among Group 1 patients (91.5 ± 3.07). Similarly, IKDC scores (77.55 ± 5.72) and Tegner Activity Scale scores (6.5 ± 5.72) were found to be better among Group 1 patients. A meta-analysis by Chang et al. [10] also summarized similar Lysholm, IKDC, and Tegner scores using suture fixation techniques as 92.2, grade A in 74%, and 6.9, respectively. Song et al. achieved a Lysholm score of 89.5 using sutures and anchors for ACL avulsion. Ahn et al. [16] reported a Lysholm score of 95.6 with suture fixation. Robert et al. documented a score of 94.2 using screws and sutures alone. In our series, we achieved a satisfactory Lysholm score of 95.4 with the arthroscopic suture pull-out technique. Seon et al. [11] compared screw and suture fixation, reporting Lysholm scores of 91.7 and 92.7, respectively.

Functional outcomes achieved in our study using suture pull-out fixation techniques were comparable to studies by Bong et al. and Yip et al. [8,17], who found suture fixation to be superior to screw fixation. Many other studies, however, found no significant functional outcome differences between these methods [11]. Tsukada et al. reported that antegrade screw fixation, while providing initial rigid fixation, caused more bony fragment comminution [18]. A meta-analysis conducted by Chang et al. concluded that there was no significant difference in functional outcomes between the two techniques. However, it was observed that screw fixation for ACL tibial avulsion fractures carries a greater risk of requiring additional surgery and implant removal [10]. The suture pull-out technique still requires more studies to refine optimal fixation strategies, such as the number of fixation points [19-23] and the number of tibial tunnels [24-27]. Based on the findings of Zheng et al. and Mutchamee et al. [27,28], the current study employed two-point fixation and two tibial tunnels, balancing simplicity with stability against displacement or malrotation. All patients attained radiological union within a mean period of eight weeks.

In this study, we addressed the gap in the literature regarding the interval between injury and surgical intervention and its impact on final functional outcomes. We found no statistically significant difference in functional outcomes between groups operated on one, two, and three weeks post-injury.

Limitations and future research

Despite these findings, our study had a limited sample size and a short follow-up duration. Future research should focus on larger, multi-center trials with longer follow-up periods to confirm these results and further explore the optimal timing for surgical intervention.

## Conclusions

Among the different arthroscopic methods, the suture pull-out technique stands out as a straightforward and effective procedure that, when correctly implemented, yields favorable clinical and functional results. Our findings indicate that the interval between injury and surgery does not impact functional outcomes when treating ACL tibial avulsions using the suture pull-out technique.
